# Determinants of Chemotherapy Convenience and Satisfaction: The Role of Symptom and Psychological Burden in Cancer Patients—A Cross-Sectional Study

**DOI:** 10.3390/healthcare14081017

**Published:** 2026-04-13

**Authors:** Evangelos C. Fradelos, Konstantinos Skampardonis, Pavlos Sarafis, Alexios Alexopoulos, Aikaterini Toska, Maria Saridi, Theocharis I. Konstantinidis, Maria Lavdaniti, Paul Cella, Athanasios Kotsakis, Filippos Koinis

**Affiliations:** 1 Laboratory of Clinical Nursing, Department of Nursing, University of Thessaly, 41500 Larissa, Greece; efradelos@uth.gr (E.C.F.); kskampard@uth.gr (K.S.); ktoska@uth.gr (A.T.); msaridi@uth.gr (M.S.); 2 Department of Nursing, University of Thessaly, 41500 Larissa, Greece; 3 First Department of Paediatrics, School of Medicine, National and Kapodistrian University of Athens, “Agia Sofia” Children’s Hospital, 11527 Athens, Greece; atosmedicals@yahoo.co.uk; 4 Department of Nursing, Hellenic Mediterranean University, 71410 Heraklion, Greece; harriskon@hmu.gr; 5 Department of Nursing, International Hellenic University, 57400 Thessaloniki, Greece; 6 FACITtrans, Ponte Vedra Beach, FL 32082, USA; pcella@facit.org; 7 Department of Medical Oncology, University Hospital of Larissa, 41500 Larissa, Greece; thankotsakis@uth.gr (A.K.); fkoinis@uth.gr (F.K.)

**Keywords:** cancer patients, chemotherapy, chemotherapy convenience, symptom burden, treatment satisfaction

## Abstract

Background: Chemotherapy-related symptom burden and psychological distress may significantly influence patients’ perceptions of treatment convenience and satisfaction, yet evidence in the Greek oncology population remains limited. Methods: A cross-sectional study was conducted among 150 cancer patients receiving chemotherapy in a tertiary hospital. Data were collected using CCSQ, ESAS-r, PHQ-2, GAD-2, and WHO-5. Pearson correlations and multiple linear regression analyses were performed. Results: Higher levels of depressive symptoms and anxiety were significantly associated with lower chemotherapy convenience and satisfaction (e.g., PHQ-2: r = −0.659 to −0.584, *p* < 0.001; GAD-2: r = −0.623 to −0.469, *p* < 0.001). Depressed mood emerged as a significant negative predictor of convenience (β = −0.39, *p* = 0.015) and concerns (β = −0.39, *p* = 0.005), while anxiety independently predicted satisfaction (β = 0.45, *p* = 0.007). Conclusions: Chemotherapy experience is strongly associated with psychological and symptom burden. These findings highlight the importance of integrating psychosocial assessment into routine oncology care.

## 1. Introduction

Over time, advances in medicine and cancer care have made cancer more of a chronic illness, although it can occasionally be a potentially fatal condition with numerous unpleasant side effects that affect the patient and their surroundings on a physical, psychological, social, and financial level [1,2,3,4]. Since cancer is seen as a multidimensional disease and treatment has a multifaceted and multilayered purpose that encompasses physical, psychological, emotional, social, and economic dimensions, the requirements of the oncological patient should be treated holistically [1,2,5,6]. Surgical procedures, semi-therapeutic treatments, and radiotherapy—all of which damage the human body—are examples of the treatment, which is frequently rather aggressive [2,3].

Chemotherapy significantly alters cancer patients’ daily lives and lifestyles, impacting various aspects of human functioning. Chemotherapy patients frequently endure a variety of unpleasant side effects because the medications’ cytotoxic effects extend beyond cancer cells to include healthy bodily parts. Physical, emotional, psychological, social, existential, and functional disorders are among the numerous systems that experience unfavorable impacts as a result [7,8]. In addition, chemotherapy-related symptoms that last for weeks and in some cases even for years can harm patients’ functioning and quality of life [9]. Researchers suggest that the onset and the intensity of symptoms are a result of the complex interaction between physical and psychological factors [9,10]. Therefore, it has been indicated that the coexistence of anxiety and depressive symptoms, along with a decline in overall psychological well-being, patients’ subjective perceptions of symptom burden, and a further decrease in their quality of life, is further decreased [11,12,13]. Understanding the complexity of the experience of chemotherapy, links and interactions between physical and psychological symptoms, as well as subjective well-being, is particularly important for providing comprehensive and personalized-centered care.

In recent years, there has been a growing emphasis on patient-reported outcomes (PROs) as key indicators of treatment efficacy and quality of care in oncology. PROs directly reflect patients’ subjective experiences by capturing specific dimensions such as symptom burden, emotional distress, and perceived impact of treatment, dimensions that are not fully captured by clinical or biomedical indicators Within this context, satisfaction with treatment and convenience, the extent to which treatment is practical and can be integrated into the patient’s daily life, are increasingly recognized as key elements of patient-centered care, as they reflect the way patients interpret and evaluate their treatment experience [14]. Convenience and satisfaction with chemotherapy are important patient-reported outcomes exceed the traditional measures of toxicity and clinical efficacy to represent the patient’s lived experience. The creation of the Chemotherapy Convenience and Satisfaction Questionnaire (CCSQ) in 2005 brought to light the inadequacy of previous quality of life measures in capturing the subjective burden related to the mode of treatment administration, practical challenges, anxiety, and overall patient satisfaction with chemotherapy [15]. These findings revealed that chemotherapy satisfaction is not just a secondary psychosocial measure, and is closely linked to adverse experiences, use of health services, and quality of life. In addition, according to a recent study, high levels of satisfaction with chemotherapy are associated with better adherence, greater involvement of patients in the care, and greater consistency throughout the treatment, especially in long-term and day-care programs [16]. However, qualitative research shows that patients appreciate the treatment’s regularity, convenience, and reduced intrusiveness in daily life, even in the face of ambivalence or doubts regarding its effectiveness [17]. Finally, in a recent Greek study, treatment satisfaction was affected by symptoms such as pain, anxiety, and fatigue. The study also highlighted the need for thorough assessment and targeted supportive care interventions [18]. Convenience and satisfaction must be key patient-reported outcomes to deliver fully complete, patient-centered oncology care.

Physical and psychological stress have an impact on patients’ perceived comfort and satisfaction with chemotherapy. The intensity and persistence of physical symptoms, such as fatigue, pain, nausea, and functional limitations, can significantly reduce the perceived ease of treatment, especially when these symptoms interfere with patients’ daily lives, autonomy, and social roles [19,20]. Concurrently, it has been shown that psychological burden, including anxiety, depressive symptoms, fear of disease progression or recurrence, and diminished mental or spiritual well-being, adversely affects the degree of overall satisfaction with chemotherapy and the subjective evaluation of the treatment experience. The fact that psychological distress may intensify the subjective perception of the severity of physical symptoms is particularly significant since it might increase the sense of intrusiveness and make therapy more difficult [21,22]. As a result, patients who experience higher levels of both physical and psychological burden are more likely to report lower levels of pleasure and perceived ease of chemotherapy, which may have a detrimental impact on treatment compliance, active participation in care, and course continuation. Therefore, the design and implementation of comprehensive, patient-centered oncology care models require an understanding of the interplay among physical burden, psychological burden, treatment satisfaction, and ease of treatment [23].

The experience of chemotherapy is a complex process and cannot be understood solely through the exploration and assessment of its physical effects. Increasing evidence suggests that patients’ perceptions of treatment are shaped by the interplay between symptom severity, psychological distress, and overall well-being. In this context, symptoms are not experienced in isolation but are also influenced by emotional factors such as anxiety and depression, which may intensify the way patients perceive and experience their condition. Moreover, a recent systematic review concludes that the experience of chemotherapy should be understood as a multidimensional concept influenced by symptom severity, fatigue, and patient-reported outcomes [24].

Therefore, treatment convenience and satisfaction should be perceived as multidimensional outcomes, reflecting not only the clinical characteristics of the treatment but also the psychological state of the patient, within the context of a more holistic, patient-centered approach to oncology care [25,26]. Current research data in Greece, regarding the Greek oncology population, is limited, as the few available studies focus mainly on the assessment of patient satisfaction with intravenous chemotherapy [18], without which these studies do not thoroughly investigate the complex and multidimensional relationship between symptom burden—especially physical and psychological—and patients’ perceived ease and satisfaction with chemotherapy.

Based on the existing literature and the identified research gap, the present study seeks to answer the following research questions: (1) What is the degree of association between physical symptom burden and psychological distress, with patients’ perceptions of convenience, concerns, and satisfaction with chemotherapy? (2) Which physical and psychological factors are significant predictors of ease, concerns, and satisfaction with chemotherapy, after controlling relevant sociodemographic and clinical variables? (3) How is overall psychological well-being related to patients’ perceived experience of chemotherapy?

Therefore, the present study aims to investigate the factors that influence the perceived convenience of chemotherapy and patient satisfaction in the Greek oncology population, with particular emphasis on physical and psychological burden.

## 2. Materials and Methods

### 2.1. Design, Sample, and Study Setting

Adult cancer patients receiving chemotherapy at a tertiary hospital’s Day Chemotherapy Unit made up the study sample for this cross-sectional study. Convenience sampling was used to pick individuals on their planned chemotherapy treatment visit. The sample size was calculated using G*Power software v.3.1 with a significance level of α = 0.05, up to 12 predictor variables, and a goal power of 0.80; the required sample size was determined to be between 70 and 90 participants. The statistical power of all major analyses was high, exceeding 95%, because the study included 150 patients overall, indicating that the sample size was sufficient to find statistically significant correlations and predictive linkages. Inclusion criteria were: (a) age ≥ 18 years, (b) confirmed diagnosis of malignancy, (c) active chemotherapy treatment on an outpatient/day basis, (d) adequate knowledge of the Greek language, and (e) ability to understand and complete the questionnaires. Patients with severe cognitive impairment, severe clinical burden that did not allow participation, or who were unable to communicate were excluded from the study. Participants represented a wide range of cancer types, such as lung, breast, and colorectal cancer. Those types were grouped into clinically and statistically homogeneous categories to ensure adequate group size and comparability of results.

### 2.2. Data Collection Tool

Data were collected using paper-based self-administered questionnaires during patients’ scheduled chemotherapy sessions. All questionnaires were administered in Greek.

**First part:** Demographic and clinical characteristics. A systematic questionnaire was used to gather the participants’ clinical and demographic details after they were enrolled. Gender (male/female), age (in years), place of residence (urban, semi-urban, or rural), marital status (single, married, divorced, widowed, or cohabiting), and living circumstance (living alone or with others) were all specifically noted. Additionally, data on educational level (illiterate, primary, secondary, post-secondary, university education, and postgraduate level) and occupational status (unemployed, private or public sector, domestic, retired) were gathered. Clinical features, cancer type, smoking status (never smoker, former smoker, current smoker), and anticancer treatment type (chemotherapy, chemotherapy plus immunotherapy, or radiotherapy) were used to characterize the sample and as confounders in statistical analyses.

**Second part:** Chemotherapy Convenience and Satisfaction Questionnaire (CCSQ). The Chemotherapy Convenience and Satisfaction Questionnaire (CCSQ) is a self-administered questionnaire developed by Yost, Hahn, and Cella within the FACIT/FACT framework to assess patients’ experience of chemotherapy [15]. It consists of 13 questions that explore three main dimensions: convenience of treatment (Convenience), concerns related to chemotherapy (Concerns), and overall satisfaction with treatment (Satisfaction). Most questions are answered on a five-point Likert-type scale (0 = “not at all” to 4 = “very much”), while some questions in the satisfaction dimension use alternative response scales, according to the tool’s instructions. The Chemotherapy Convenience and Satisfaction Questionnaire (CCSQ) is a self-administered questionnaire developed by Yost, Hahn, and Cella within the FACIT/FACT framework to assess patients’ experience of chemotherapy, and it assesses three aspects of treatment: convenience (Convenience), chemotherapy-related concerns (Concerns), and general treatment satisfaction (Satisfaction) [10]. According to the instrument’s instructions, most items are rated on a five-point Likert-type scale (0 = “not at all” to 4 = “very much”), although some items in the satisfaction dimension use alternative response formats. The psychometric properties of the Greek version of CCSQ, as presented in this study, are satisfactory. Internal consistency ranged from acceptable to high across the scale dimensions (Cronbach’s α = 0.766–0.861; McDonald’s ω up to 0.882).

**Third part:** The Edmonton Symptom Assessment System–Revised (ESAS-r) was used to evaluate patients’ symptom burden. This scale assesses nine primary physical and psychological symptoms such as pain, exhaustion, sleepiness, nausea, loss of appetite, dyspnea, low mood, anxiety, and general well-being. Those symptoms are assessed on a scale of 0 to 10. Apart from general well-being, where higher scores imply better condition, higher values indicate greater intensity of each symptom. The ESAS-r is a well-known instrument, and it is frequently used in oncology therapy and research. Cronbach’s α has been reported to range from approximately 0.70 to 0.88 in oncology populations [27,28].

**Fourth part:** To evaluate psychological distress and well-being, the following measures were used. The Patient Health Questionnaire-2 (PHQ-2) [29,30] was used to measure depressive symptomatology. It consists of two questions about the presence of anhedonia and depression during the previous two weeks, with responses on a four-point scale (0 = “not at all” to 3 = “nearly every day”). The Generalized Anxiety Disorder-2 (GAD-2) [30,31], which has two items and the same rating scale, was used to measure anxiety. Higher total scores indicate higher levels of anxiety symptomatology. Finally, psychological well-being was assessed with the World Health Organization–Five Well-Being Index (WHO-5), a short instrument of five positively worded questions that assess subjective well-being over the past two weeks. Each question is scored on a six-point scale (0–5), with total scores converted to a scale of 0–100, where higher values correspond to better psychological well-being [31,32]. The PHQ-2 and GAD-2, despite their brevity, have demonstrated acceptable internal consistency, with reported Cronbach’s α values ranging from approximately 0.70 to 0.85. The WHO-5 Well-Being Index has shown strong reliability in various populations, including Greek samples, with Cronbach’s α values typically ranging from 0.84 to 0.91.

The average time required to complete the questionnaire was approximately 20 min, indicating a low to moderate respondent burden.

### 2.3. Statistical Analysis

Statistical analysis of the data was performed using IBM SPSS Statistics (version 26) and JASP 0.18.3.0. The sociodemographic and clinical features of the sample, as well as the factors of ease, worries, and satisfaction with chemotherapy, symptom load, and psychological burden, were first presented using descriptive statistics. Whereas continuous data were shown as means (M) and standard deviations (SD), categorical variables were shown as absolute frequencies (n) and percentages (%). Confirmatory factor analysis (CFA) was used to verify the structural validity of the Chemotherapy Convenience and Satisfaction Questionnaire (CCSQ), Model fit in confirmatory factor analysis was evaluated using the Comparative Fit Index (CFI), Tucker–Lewis Index (TLI), Root Mean Square Error of Approximation (RMSEA), and Standardized Root Mean Square Residual (SRMR). as well as the χ^2^/df ratio. To evaluate the internal consistency, Cronbach’s alpha was calculated. The distribution of continuous variables was assessed using Kolmogorov–Smirnov tests, as well as inspection of skewness and kurtosis values. The results indicated acceptable approximation to normality, supporting the use of parametric statistical tests. Pearson correlations were used to examine the relationships between the scales that were used in this study, accounting for potential confounding variables like gender, age, occupational status, education level, and cancer type. Finally, in the regression analyses, symptom burden and psychological variables were treated as independent variables (predictors), while the CCSQ dimensions (Convenience, Concerns, and Satisfaction) were defined as dependent variables. Models were additionally controlled for sociodemographic variables such as gender, age, occupational status, education level, and cancer type. *p* < 0.05 was set as the threshold for statistical significance.

### 2.4. Ethical Considerations

The study was conducted in accordance with the Declaration of Helsinki and was approved by the Institutional Review Board of the University Hospital of Larissa (approval reference number: 35/21/19-12-2024). All participants were informed about the purpose and procedures of the study, as well as their right to withdraw at any time without consequences. Written informed consent was obtained from all participants before data collection.

The study was reported in accordance with the STROBE Guidelines (STROBE Checklist in Supplementary File).

## 3. Results

The study sample consisted of 150 patients, the majority of whom were male, married, and retired, with a significant proportion having a low educational level and residing in urban areas. The most common form of cancer was lung cancer, while half of the participants were active smokers. Chemotherapy was the main therapeutic approach, either as monotherapy or in combination with immunotherapy (Table 1).

For the linguistic and cultural adaptation of the tool, the standardized translation and back-translation process recommended by the FACIT/FACT organization was followed, including independent translation, version consolidation, back-translation, and final expert evaluation, to ensure conceptual and linguistic equivalence. The three-factor structure of the tool was well confirmed by the confirmatory factor analysis (CFA) based on the psychometric study results, with fit indices deemed satisfactory (CFI = 0.950, TLI = 0.935, RMSEA = 0.065, SRMR = 0.051). The Chi-square to degrees of freedom ratio, or χ^2^/df, index was computed to be 1.63 (χ^2^ = 83.013, df = 51), which is below the allowed limit and suggests that the model fits the data well. Each question’s contribution to the corresponding factors was confirmed by the statistically significant and satisfactorily ranging factor loadings of the individual questions. The scale’s overall reliability is regarded as moderate to satisfactory, although internal consistency revealed variances amongst the factors, with the third factor exhibiting strong reliability (Cronbach’s α = 0.861, ω = 0.882). Furthermore, the scale’s general internal consistency is deemed adequate by the unidimensional reliability index (Cronbach’s α = 0.766). The results confirm the tool’s structural validity and psychometric suitability in the sample under study.

Overall, participants reported moderate levels of ease and concerns and relatively high satisfaction with chemotherapy. with the satisfaction dimension presenting the highest mean value (M = 72.55, SD = 19.91). Regarding the symptom burden (ESAS-r). fatigue (M = 4.91. SD = 1.55) and anxiety (M = 4.34. SD = 1.69) emerged as the most intense symptoms. while general well-being showed moderate levels (M = 4.70. SD = 1.66). Finally, the psychological burden indicators demonstrated mild to moderate symptoms of depression and anxiety. with mean values of 2.27 (SD = 1.45) for PHQ-2 and 2.09 (SD = 1.80) for GAD-2 (Table 2).

Partial correlation analyses revealed statistically significant associations between all CCSQ dimensions and psychological and symptomatic variables. Higher levels of ease and satisfaction with treatment were moderately to strongly associated with lower levels of depressive and anxiety symptoms (indicatively PHQ-2: r = −0.659 to −0.584 and GAD-2: r = −0.623 to −0.469, all *p* < 0.001), as well as with reduced overall symptom burden, particularly fatigue and pain. In contrast, well-being, as assessed with the WHO-5, showed strong positive correlations with ease, worries, and satisfaction (r = 0.639–0.656, *p* < 0.001), suggesting that a more positive experience of chemotherapy treatment is closely associated with higher levels of psychological well-being (Table 3).

The results of the multiple linear regressions showed that the three models for the dimensions Convenience, Concerns, and Satisfaction presented a statistically significant overall fit, explaining a large percentage of the variance in the corresponding dimensions of the CCSQ (R^2^ = 0.651 for Convenience, R^2^ = 0.741 for Concerns, and R^2^ = 0.558 for Satisfaction, all *p* < 0.001). Depressed mood (ESAS-r) emerged as a consistent and statistically significant negative predictor of both. Ease and worries from chemotherapy, while pain and fatigue were independently associated with increased levels of worries. In contrast, for the Satisfaction dimension, anxiety as assessed by the GAD-2 was the only statistically significant independent predictor. These findings highlight the role of psychological and symptomatic burden in patients’ perception of chemotherapy treatment (Table 4).

## 4. Discussion

The purpose of this study was to investigate the factors that shape the ease and satisfaction with chemotherapy in the Greek oncology population, with an emphasis on the role of physical and psychological burden.

To examine treatment satisfaction, the CCSQ was used. The CCSQ was used for the first time that it was used in the Greek context; thus, it was evaluated for its factor structure and reliability. Results of the confirmatory factor analysis (CFA) indicated a satisfactory to good fit between the proposed model and the empirical data is indicated by the model fit indices (CFI = 0.950, TLI = 0.935, RMSEA = 0.065, SRMR = 0.051, χ^2^/df = 1.63). Our results agree with the findings from the study of Yost, Hahn, and Cella (2005) [15], wherein exploratory factor analysis and Rasch analysis revealed a multidimensional structure of the CCSQ consisting of three distinct subscales: concerns, convenience, and satisfaction. In addition, our study demonstrated good reliability (Cronbach’s α = = 0.766), levels that were the same as those reported in the previous study (α = 0.80–0.86). This finding regarding the psychometric properties of the scale supports its use in diverse populations and across different cultural settings. Conversely, the scale’s moderate to satisfactory overall reliability and the observed variance in reliability between variables represent a finding that warrants careful interpretation. In the current research, a slightly lower overall reliability was observed. This finding may be due to variations in the sample, treatment phase, or cultural factors that affect perceptions of convenience and satisfaction with treatment when compared to the study by Yost et al. [15], where all reliability indices were high. Finally, the results of the present study support that CCSQ is a valid instrument that can be used to examine patient experience with treatment. The high-level agreement of this study with the original proposed structure and reliability indicates that the instrument can be used in future studies in Greece to assess patients’ experience with chemotherapy.

The present study provided a glimpse of patients’ chemotherapy convenience and satisfaction by using CCSQ. According to our results, a low mean value was observed on the convenience subscale; this finding suggests that there is an increased practical and functional burden of treatment. This finding opposes other studies that examine treatment convenience and satisfaction. For instance, in a study conducted evaluating treatment satisfaction in the context of randomized phase III trials in metastatic colorectal cancer, the XELOX regimen was associated with significantly higher perceived convenience and fewer hospital days compared to FOLFOX-6. According to this research, reducing the need for lengthy IV infusions and administering capecitabine orally are important elements in enhancing patient satisfaction [33]. Furthermore, data from studies using capecitabine in conjunction with cisplatin for metastatic head and neck cancers revealed a decline in treatment convenience without a significant decline in overall satisfaction, indicating that the practical burden rises with treatment duration and toxicity [34].

Another interesting finding of our study is that despite the low convenience, our sample’s mean satisfaction score (72.55) is nevertheless similar to that of patients getting regimens based on both FOLFOX and XELOX or combinations with targeted medicines. Even when toxicity or treatment burden increased, patient satisfaction remained high in randomized trials comparing FOLFOX-bevacizumab with alternative regimens [35]. A possible explanation of this finding is that patients value more the perceived efficacy, trust the physician and treatment strategy, and relationship with the care team rather than practical convenience. In addition, the moderate level of concerns (Concerns: 58.89) that were observed in our study supports the idea that the psychological and functional burden of chemotherapy remains a crucial aspect of the patient’s experience, even in treatment considered to be acceptable or “convenient.”

The results of this study provide a complex and multidimensional picture of patients’ experiences with chemotherapy, highlighting the role that psychological distress and symptom burden play in influencing perceived convenience, concerns, and treatment satisfaction, according to the Chemotherapy Convenience and Satisfaction Questionnaire (CCSQ). Significant correlations between all features of the CCSQ and indicators of depression, anxiety, overall well-being, and core cancer-related symptoms demonstrate the close interaction between physical and psychological aspects of the chemotherapy experience [36,37].

The present findings could be interpreted and further evaluated in the context of patient-reported outcomes (PROs) and symptom cluster theory. Patients evaluate treatment not only based on clinical outcomes, but also through the cumulative effect of co-occurring symptoms and emotional states, as captured through self-reported data, demonstrating a strong association between psychological distress, symptom severity, and chemotherapy experience [14,38].

According to the symptom cluster theory, symptoms in cancer patients do not operate independently but tend to coexist and interact, forming complex, dynamic clusters that collectively influence patients’ perceptions of treatment burden and quality of life [39]. The attenuation of the effects of individual symptoms observed in multivariate models may reflect the interdependent relationship between physical and psychological symptoms, with psychological distress—and particularly depressive symptomatology—emerging as a central organizing factor [26].

This finding highlights the importance of approaching the chemotherapy experience as a holistic construct, where psychological and physical dimensions constitute interdependent components. Furthermore, it highlights the value of integrating PROs into daily oncology practice, as they enable a more comprehensive understanding of patients’ experiences, contribute to improved symptom detection and assessment, and are better associated with clinical outcomes, including prolonged survival and reduced healthcare utilization [17]. The discovery that depressed symptomatology demonstrated the strongest relationships with all CCSQ characteristics, whether using specific mood indicators (ESAS-r) and short screening measures (PHQ-2), is particularly significant. Furthermore, low mood was identified as an independent predictor of perceived treatment ease and chemotherapy-related concerns in multivariate regression analyses, after adjustment for multiple sociodemographic variables. This outcome is consistent with global research that indicates melancholy can be a mediating mechanism that converts physical stress into a negative evaluation of the therapeutic encounter [37,40,41].

Several symptoms did not sustain statistical significance in regression models, although symptoms like pain, exhaustion, appetite loss, and anxiety showed substantial bivariate relationships with CCSQ dimensions. The symptom cluster theory, which holds that cancer symptoms frequently coexist and have similar underlying processes, can be used to analyze this pattern. While several physical symptoms were associated with chemotherapy experience in the initial analyses, many of these relationships became weaker in the regression models. This may indicate that physical symptoms and psychological distress are closely interrelated, with depressive symptoms showing the most consistent association with patients’ perceptions of treatment [42,43].

The discovery that anxiety retained a statistically significant impact on treatment satisfaction despite not being an independent predictor for all CCSQ characteristics is also notable. This implies that anxiety, which is associated with uncertainty and worry about the course of the illness, may have a bigger influence on the cognitive and emotional evaluation of treatment [9]. Lastly, all CCSQ categories showed high positive associations with subjective well-being as measured by the WHO-5 and the related ESAS-r indices; however, in multivariate models, subjective well-being lost its independent predictive significance. This result aligns with previous research indicating that well-being primarily reflects the combined effects of psychological state and symptom burden rather than acting as a causal factor [25].

Despite the important findings of this study, several limitations should be acknowledged. The cross-sectional design does not allow for causal inferences, but only the possibility of identifying associations between symptom burden, psychological distress, and patients’ perceptions of chemotherapy. Furthermore, the use of a convenience sample, drawn from a single hospital setting, limits the generalizability of the results, as patients’ experiences in other healthcare settings or regions may differ. Reliance on self-report data may also have introduced response biases, as patients’ responses could have been influenced by their emotional state at the time of participation in the study.

Furthermore, although validated instruments were used, the use of short screening tools such as the PHQ-2 and GAD-2 may not fully reflect the complexity of patients’ psychological distress. Finally, important clinical variables, such as disease stage or treatment history, were not included in the study, and this absence may have influenced the interpretation of the findings. Therefore, the use of longitudinal designs and more diverse samples from future studies is needed to provide a deeper and more comprehensive understanding of the chemotherapy experience.

## 5. Conclusions

The study’s findings indicated that patients’ experiences with chemotherapy are influenced by a wider psychosocial environment rather than just treatment features or physical harm. A fundamentally person-centered approach to chemotherapy care might be reinforced by the systematic identification and early treatment of depression and anxiety, which could significantly reduce the perceived burden of treatment, improve patients’ treatment experience, and support the delivery of more patient-centered oncology care.

## Figures and Tables

**Table 1:** Sociodemographic and Clinical Characteristics of the Study Sample (N = 150).

Variable	Category	n	%
Gender	Male	96	64.0
	Female	54	36.0
Age			67.92 (10.9)
Marital Status	Single	12	8.0
	Married	101	67.3
	Divorced	4	2.7
	Widowed	28	18.7
	Cohabiting	5	3.3
Living Alone	Yes	26	17.3
	No	124	82.7
Occupation	Unemployed	2	1.3
	Private sector	25	16.7
	Public sector	9	6.0
	Household	10	6.7
	Pensioner	104	69.3
Education Level	Illiterate	50	33.3
	Primary education	58	38.7
	Junior high school	11	7.3
	High school	13	8.7
	Technological education	1	0.7
	University	11	7.3
	MSc	6	4.0
Area of Residence	Urban	85	56.7
	Semi-urban	33	22.0
	Rural	32	21.3
Cancer Type	Breast	29	19.3
	Lung	64	42.7
	Gastrointestinal	34	22.7
	Other	23	15.3
Smoking Status	Past smoker	45	30.0
	Current smoker	75	50.0
	Never smoker	30	20.0
Type of Treatment	Chemotherapy	89	59.3
	Chemotherapy + Immunotherapy	61	40.7

Data are presented as absolute frequencies (n) and percentages (%).

**Table 2:** Descriptive Statistics of Treatment Satisfaction, Symptom Burden, and Psychological Distress Measures.

Variable	Mean	Standard Deviation	Minimum	Maximum
*Chemotherapy Convenience and Satisfaction Questionnaire (CCSQ)*				
Convenience	41.22	15.34	8.33	83.33
Concerns	58.89	14.33	15.00	90.00
Satisfaction	72.55	19.91	27.08	100.00
*Edmonton Symptom Assessment System Revised (ESAS-r)*				
Pain	2.77	1.66	0.00	8.00
Fatigue	4.91	1.55	1.00	9.00
Drowsiness	2.64	1.49	0.00	7.00
Nausea	3.09	1.33	0.00	7.00
Loss of appetite	3.73	1.64	0.00	9.00
Difficulty breathing	2.76	2.11	0.00	7.00
Depressed mood	3.34	2.12	0.00	8.00
Anxiety	4.34	1.69	0.00	9.00
Well-being	4.70	1.66	1.00	9.00
*Psychological Burden*				
PHQ-2 Depression	2.27	1.45	0.00	6.00
GAD-2 Anxiety	2.09	1.80	0.00	12.00
WHO-Wellbeing 5	42.89	18.96	4.00	80.00

**Table 3:** Pearson’s Correlations between CCSQ Dimensions and Symptom and Psychological Variables.

Variable	Convenience (r)	Concerns (r)	Satisfaction (r)
PHQ-2 Depression	−0.659 ***	−0.645 ***	−0.584 ***
GAD-2 Anxiety	−0.558 ***	−0.623 ***	−0.469 ***
WHO-5 Well-being	0.639 ***	0.654 ***	0.656 ***
Pain	−0.405 ***	−0.587 ***	−0.397 ***
Fatigue	−0.534 ***	−0.632 ***	−0.500 ***
Drowsiness	−0.441 ***	−0.440 ***	−0.384 ***
Nausea	−0.213 *	−0.208 *	−0.297 ***
Loss of appetite	−0.529 ***	−0.538 ***	−0.522 ***
Difficulty breathing	−0.186 *	−0.332 ***	−0.160
Depressed mood (ESAS-r)	−0.704 ***	−0.700 ***	−0.558 ***
Anxiety (ESAS-r)	−0.677 ***	−0.715 ***	−0.540 ***
Well-being (ESAS-r)	−0.609 ***	−0.668 ***	−0.595 ***

Pearson’s correlations controlled for gender, age, occupational status, education level, and cancer type. * *p* < 0.05; *** *p* < 0.001.

**Table 4:** Multiple Linear Regression Analysis with Chemotherapy Concerns as the Dependent Variable and Edmonton Symptom Assessment System Revised (ESAS-r), PHQ2, GAD2, and WHO Wellbeing 5 as Independent Variables (Predictors).

***Model for the dimension of convenience***						
Predictor	B	SE	β	t	*p*	95% CI
Intercept	81.91	17.10	—	4.79	<0.001	48.07, 115.75
Pain	0.39	0.74	0.04	0.52	0.601	−1.08, 1.85
Fatigue	−0.54	1.10	−0.06	−0.49	0.624	−2.71, 1.63
Drowsiness	−1.40	1.01	−0.14	−1.38	0.169	−3.40, 0.60
Nausea	1.11	0.93	0.10	1.20	0.234	−0.72, 2.94
Loss of appetite	−1.21	0.97	−0.13	−1.25	0.214	−3.13, 0.71
Difficulty breathing	1.03	0.65	0.14	1.58	0.117	−0.26, 2.32
Depressed mood (ESAS-r)	−2.82	1.14	−0.39	−2.47	**0.015**	−5.08, −0.56
Anxiety (ESAS-r)	−2.03	1.09	−0.22	−1.86	0.066	−4.19, 0.13
Well-being (ESAS-r)	−1.10	1.38	−0.12	−0.80	0.426	−3.83, 1.64
PHQ-2 Depression	−1.57	1.24	−0.15	−1.26	0.209	−4.02, 0.88
GAD-2 Anxiety	1.47	0.90	0.17	1.64	0.104	−0.31, 3.25
R = 0.807; R^2^ = 0.651; Adjusted R^2^ = 0.551; F(33,115) = 6.51, *p* < 0.001; RMSE = 10.28.						
**Model for the dimension of concerns**						
Predictor	B	SE	β	t	*p*	95% CI
Intercept	104.95	13.77	—	7.62	<0.001	77.69, 132.21
Pain	−1.85	0.60	−0.21	−3.10	**0.002**	−3.03, −0.67
Fatigue	−2.77	0.89	−0.30	−3.11	**0.002**	−4.53, −1.01
Drowsiness	0.04	0.82	0.00	0.05	0.959	−1.58, 1.66
Nausea	0.80	0.75	0.07	1.08	0.283	−0.68, 2.29
Loss of appetite	−0.08	0.78	−0.01	−0.11	0.916	−1.62, 1.46
Difficulty breathing	−0.39	0.53	−0.06	−0.74	0.462	−1.43, 0.66
Depressed mood (ESAS-r)	−2.66	0.92	−0.39	−2.89	**0.005**	−4.47, −0.85
Anxiety (ESAS-r)	−1.67	0.88	−0.20	−1.91	0.059	−3.41, 0.06
Well-being (ESAS-r)	−0.10	1.11	−0.01	−0.09	0.932	−2.29, 2.10
PHQ-2 Depression	0.29	1.00	0.03	0.29	0.775	−1.68, 2.26
GAD-2 Anxiety	−0.21	0.72	−0.03	−0.29	0.776	−1.63, 1.21
WHO-5 Well-being	−0.06	0.10	−0.09	−0.68	0.499	−0.25, 0.12
R = 0.861; R^2^ = 0.741; Adjusted R^2^ = 0.667; F(33,115) = 9.97, *p* < 0.001; RMSE = 8.28.						
***Model for the dimension of satisfaction***						
Predictor	B	SE	β	t	*p*	95% CI
Intercept	67.80	24.99	—	2.71	0.008	18.29, 117.30
Pain	0.59	1.08	0.05	0.55	0.586	−1.56, 2.74
Fatigue	−1.15	1.61	−0.09	−0.71	0.479	−4.34, 2.05
Drowsiness	0.04	1.48	0.00	0.03	0.980	−2.90, 2.97
Nausea	0.19	1.35	0.01	0.14	0.889	−2.49, 2.87
Loss of appetite	−1.41	1.42	−0.12	−1.00	0.321	−4.22, 1.39
Difficulty breathing	1.82	0.95	0.19	1.91	0.059	−0.07, 3.70
Depressed mood (ESAS-r)	−0.42	1.67	−0.04	−0.25	0.804	−3.72, 2.90
Anxiety (ESAS-r)	−1.38	1.59	−0.12	−0.87	0.387	−4.54, 1.77
Well-being (ESAS-r)	−2.66	2.01	−0.22	−1.32	0.188	−6.65, 1.32
PHQ-2 Depression	−1.51	1.81	−0.11	−0.84	0.405	−5.10, 2.07
GAD-2 Anxiety	0.48	0.17	0.45	2.76	**0.007**	0.13, 0.82
R = 0.747; R^2^ = 0.558; Adjusted R^2^ = 0.431; F(33,115) = 4.39, *p* < 0.001; RMSE = 15.02.						

**Note:** (1) R represents the correlation between observed and predicted values; R2 indicates the proportion of variance explained by the model; Adjusted R2 accounts for the number of predictors included; F reflects the overall significance of the model; RMSE represents the average prediction error. (2) Models were additionally controlled for sociodemographic variables such as gender, age, occupational status, education level, and cancer type.

## Data Availability

The data presented in this study are available on request from the corresponding author because the dataset includes sensitive health-related information.

## Supplementary Materials

The following supporting information can be downloaded at: https://www.mdpi.com/article/10.3390/healthcare14081017/s1, Supplementary Materials File: STROBE Checklist.
